# 

**Published:** 1883-11

**Authors:** 


### BOOKS AND PAMPHLETS RECEIVED.

### BOOKS.

HISTORY OF TUBERCULOSIS. From Dr. Arnold Spina, translated by Sattler. Cincinnati: R. Clark & Co.

LETTSOMIAN LECTURES ON VALVULAR HEART DISEASE. Sansom. Chicago: W. T. Keener. Philadelphia: Blakiston, Son & Co.

TREATMENT OF DISEASES OF CHILDREN. Goodwin. New York: C. H. Goodwin.

EXCISION OF THE KNEE-JOINT. Fenwick. Montreal: Dawson Bros.

REPORT OF THE SECRETARY OF WAR. 1880. Vol. IV.

RECORD FOR THE SICK ROOM. Blakiston, Son & Co. Chicago: W. T. Keener.

TEXT-BOOK OF PATHOLOGICAL ANATOMY. Ziegler. New York: W. M. Wood & Co. Chicago: W. T. Keener.

GRAY'S ANATOMY. Tenth Edition. Philadelphia: Henry C. Lea's Son & Co. Chicago: Jansen, McClurg & Co.

SOELBERG WELLS ON THE EYE. Philadelphia: Henry C. Lea's Son & Co. Chicago: Jansen, McClurg & Co.

HAND-BOOK FOR HOSPITALS. New York: G. P. Putnam's Sons. Chicago: Jansen, McClurg & Co.

INDEX CATALOGUE OF THE LIBRARY OF THE SURGEON-GENERAL'S OFFICE. Washington: Government Printing Office.

PRINCIPLES AND PRACTICE OF SURGERY. Agnew. Philadelphia: Lippincott & Co. Chicago: S. A. Maxwell & Co.

HEWITT'S DISEASES OF WOMEN. Sims. 2 Vols. New York: Birmingham & Co.

GENITO-URINARY DISEASES AND SYPHILIS. Otis. New York: Birmingham & Co.

PRACTICAL MANUAL OF DISEASES OF CHILDREN. Ellis. New York: Birmingham & Co.

AITKEN'S HAND-BOOK OF TREATMENT. New York: Birmingham & Co.

WOOD'S MATERIA MEDICA AND TOXICOLOGY. Fifth Edition. J. B. Lippincott & Co.

QUIZ' COMPENDIUM. MATERIA MEDICA. Philadelphia: P. Blakiston Son & Co.

THE TREATMENT OF WOUNDS. Pilcher. New York: Wm. Wood & Co., Medical Library, August. Chicago: W. T. Keener.

CLASSIFICATION, DIAGNOSIS, AND TREATMENT OF INSANITY. Spitzka. New York: Birmingham & Co.

DISEASES OF THE EAR. Pomeroy. New York: Birmingham & Co.

STUDENTS' MANUAL OF CHEMISTRY. Wetthaus. New York: Wm. Wood & Co.

A GUIDE TO THE MEDICAL STUDENT IN EUROPE. Hun. New York: Wm. Wood & Co.

A TREATISE ON THERAPEUTICS, COMPRISING MATERIA MEDICA AND TOXICOLOGY, WITH ESPECIAL REFERENCE TO THE PHYSIOLOGICAL ACTION OF DRUGS TO CLINICAL MEDICNE. By H. C. Wood. Philadelphia: J. B. Lippincott & Co. Chicago: S. A. Maxwell & Co.

The fifth edition of this excellent book follows fast upon the fourth. The latest addtions to the Pharmacopœia are incorporated, the whole work revised and enlrrged, keeping well abreast of the most recent therapeutic literature of the present day. A compact volume of over 700 pages.

PAMPHLETS.

Anomalies in the Circulatory Apparatus.—Winslow.

Erysipelas, Purpura, and Gangrene.—Meachem.

Remarks on Hydrophobia.—Dulles.

Recent Advances in the Surgery of the Urinary Organs.—Harrison.

General Paralysis of the Insane.—McFarland.

A Rectal Obturator.—Prince.

The Bead Suture.—Prince.

Heart Puncture and Heart Suture.—Roberts.

Treatment of Opium Addiction—.Mattison.

Clinical Notes on Opium Addiction.—Mattison.

The Curability of Opium Addiction.—Mattison.

Neurotic Pyrexia and Opium Addiction.—Mattison.

Treatment of Bubo.—Lydston.

Hypodermic Treatment of Syphilis.—Lagorio.

Removal of Both Ovaries.—Etheridge.

Post-Partum Malarial Fever.—Burdick.

Exterge Oculum.—Eggemann.

Antiseptic Remedies in the Treatment of Diseases of the Eye and Ear.—Holmes.

Report of Illinois State Board of Health, June, 1883.

